# Theoretical Study of Monolayer and Double-Layer Waveguide Love Wave Sensors for Achieving High Sensitivity

**DOI:** 10.3390/s17030653

**Published:** 2017-03-22

**Authors:** Shuangming Li, Ying Wan, Chunhai Fan, Yan Su

**Affiliations:** School of Mechanical Engineering, Nanjing University of Science and Technology, Nanjing 210094, China; wanying@njust.edu.cn

**Keywords:** love wave, sensors, SiO_2_, monolayer, double-layer, waveguide

## Abstract

Love wave sensors have been widely used for sensing applications. In this work, we introduce the theoretical analysis of the monolayer and double-layer waveguide Love wave sensors. The velocity, particle displacement and energy distribution of Love waves were analyzed. Using the variations of the energy repartition, the sensitivity coefficients of Love wave sensors were calculated. To achieve a higher sensitivity coefficient, a thin gold layer was added as the second waveguide on top of the silicon dioxide (SiO_2_) waveguide–based, 36 degree–rotated, Y-cut, X-propagating lithium tantalate (36° YX LiTaO_3_) Love wave sensor. The Love wave velocity was significantly reduced by the added gold layer, and the flow of wave energy into the waveguide layer from the substrate was enhanced. By using the double-layer structure, almost a 72-fold enhancement in the sensitivity coefficient was achieved compared to the monolayer structure. Additionally, the thickness of the SiO_2_ layer was also reduced with the application of the gold layer, resulting in easier device fabrication. This study allows for the possibility of designing and realizing robust Love wave sensors with high sensitivity and a low limit of detection.

## 1. Introduction

Surface acoustic wave (SAW) devices have been used in various sensing applications such as temperature [1], stress [2], gas [3], chemical and biological sensing [4] fields. The SAW sensor is a piezoelectric mass sensor which is sensitive to the mass loading on its surface [5]. The mass loading can influence the propagation of acoustic waves, as detected from changes in the velocity, phase, and amplitude. Thus, SAW sensors can be utilized to detect biomarkers which can be captured specifically by the modified surface. Owing to their high sensitivity, low cost, ease of integration with electronic circuits and possibility of real-time monitoring, SAW biosensors have been widely used for the bio-detection of proteins, DNA and cells [6,7,8]. With these advantages, SAW biosensors have a great potential in clinical diagnosis, especially in point-of-care testing for portable sensing applications.

There are various types of SAWs, such as the Rayleigh wave [9], the surface skimming bulk wave (SSBW) [10], and the Love wave [11]. The Rayleigh wave, which propagates with an elliptical particle displacement, presents a vibration component in the surface normal direction. The damping of the water causes a high energy loss leaking into the liquid, and limits the application in liquid detection. SSBW is a kind of shear horizontal SAW (SH-SAW), which can be used for liquid detection. However, its waves are not confined to the surface, resulting in a high insertion loss and a low mass loading sensitivity.

The Love wave sensor, also named the guided SH-SAW sensor, is a favored device for liquid phase applications [12,13,14]. This device has a low velocity waveguide layer on the substrate. The waveguide layer reduces power consumption and increases sensitivity by confining most of the wave energy in this layer.

Silicon dioxide (SiO_2_) is a commonly used waveguide material for the Love wave sensors due to its chemical stability, in comparison to other polymer materials such as polymethylmethacrylate (PMMA) and positive photoresists [11,15,16,17]. Among various piezocrystals, 36 degree–rotated Y-cut X-propagating lithium tantalate (36° YX LiTaO_3_) shows a high electromechanical coupling coefficient (K^2^) and low power consumption [16,18]. The propagation of the acoustic waves in 36° YX-LiTaO_3_ is shear horizontal, showing great potential in liquid detection [19]. When a SiO_2_ layer as the waveguide is added, the waves transform to Love waves, which are more sensitive to the mass loading.

According to the literature [16,20,21,22], the wavelengths of the 36° YX-LiTaO_3_ Love wave sensors usually range from 20 μm to 40 μm, with center frequencies from about 210–105 MHz. Although a higher center frequency can increase the mass loading sensitivity, it can also cause difficulties in circuit design and testing, thereby creating a high noise level. Thus, the most widely used method to increase the sensitivity is to optimize the waveguide layer thickness. However, the optimal waveguide thickness corresponding to the highest sensitivity is much larger than that having the lowest insertion loss [11]. High insertion loss decreases the signal-to-noise ratio, and thus worsens the limit of detection. It also suffers from a disadvantage to the miniaturization of portable detection systems, because of the high power consumption.

Double-layer waveguide presents an effective method to improve the performance of Love wave sensors [23,24]. By adding an ultra-low-velocity layer on the SiO_2_ waveguide layer, the Love wave velocity could be further decreased and more wave energy may be trapped in the waveguide layer. Thus, the Love wave sensor can be made more sensitive to mass loading on the surface. Table 1 shows the important parameters of SiO_2_, gold (Au) and 36° YX LiTaO_3_. Compared with SiO_2_, Au has a much lower velocity and the potential to be used as the second waveguide layer of Love wave sensors. Besides, it also shows stable physicochemical properties and biocompatibility for biomolecule self-assembly [25].

In this work, a thin Au layer is added as the second waveguide layer on top of the SiO_2_-based 36° YX LiTaO_3_ Love wave sensor in order to achieve a higher sensitivity coefficient and a thinner optimal waveguide thickness. We have theoretically analyzed both the monolayer and double-layer waveguide Love wave sensors. The methods to solve the displacements, energy distributions and sensitivity coefficients of these two types of Love wave sensors are discussed. The Love wave velocity and energy distributions are calculated and the optimal thicknesses of waveguide layers are also obtained.

## 2. Theoretical Analysis

### 2.1. Love Wave Propagation Equation

**Figure 1:**
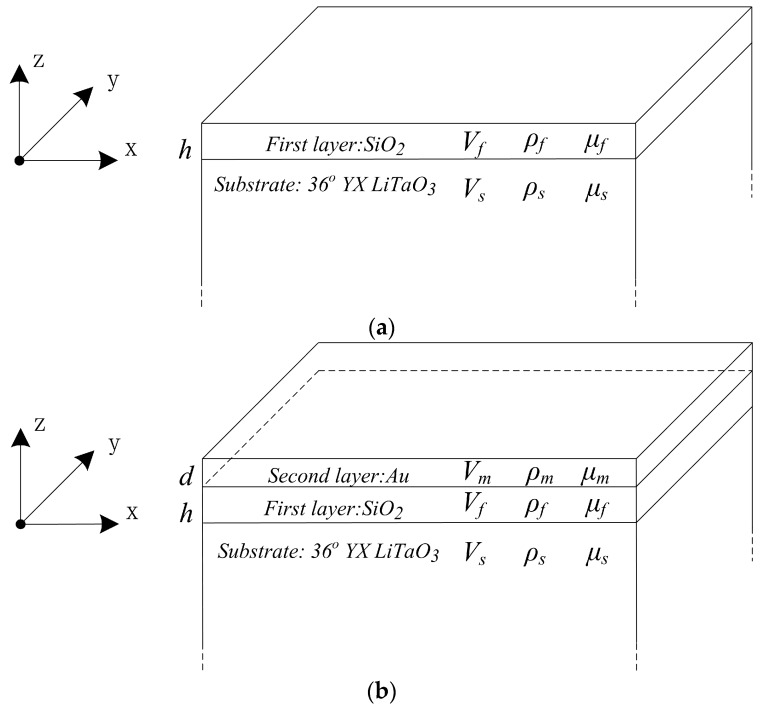
Diagram of the Love wave sensor: (**a**) monolayer; (**b**) double-layer.

As the thickness of the substrate is much larger than that of the waveguide layer, the Love wave device is generally regarded as a semi-infinite half-space. Figure 1 shows the diagrams of the monolayer and double-layer waveguide Love wave sensor structures. The coordinate system is built up, and the original point is located on the boundary surface of the substrate and waveguide layer.

The *x*-axis is the longitudinal direction that the Love wave is propagating along; the *y*-axis is the shear horizontal direction of the particle vibration; and the *z*-axis is the normal direction of the device surface. Because the particle displacement is shear horizontally polarized, the piezoelectric substrate can be simplified as an isotropic or highly symmetrical anisotropic material.

The symbols h and d are the thicknesses of the first (SiO_2_) and second (Au) waveguide layers. The symbols $V_{S}$, $V_{f}$ and $V_{m}$ represent the shear velocities, while $\rho_{S}$, $\rho_{f}$ and $\rho_{m}$ stand for the densities of the respective material. The shear moduli of the substrate (36° YX LiTaO_3_), first (SiO_2_) and second (Au) waveguide layers are represented by $\mu_{S}$, $\mu_{f}$ and $\mu_{m}$, respectively.

The Love wave propagation equations of the double-layer structure can be defined as shown in Equations (1)–(3). The monolayer Love wave propagation equations are comprised of Equations (1) and (2) [26,27].

(1) $$(\frac{\partial^{2}}{\partial x^{2}}+\frac{\partial^{2}}{\partial z^{2}}+\frac{\omega^{2}}{V_{f}^{2}})u_{yf}=0$$

(2) $$(\frac{\partial^{2}}{\partial x^{2}}+\frac{\partial^{2}}{\partial z^{2}}+\frac{\omega^{2}}{V_{S}^{2}})u_{yS}=0$$

(3) $$(\frac{\partial^{2}}{\partial x^{2}}+\frac{\partial^{2}}{\partial z^{2}}+\frac{\omega^{2}}{V_{m}^{2}})u_{ym}=0$$

The symbol ω is the radian frequency of the Love wave; $u_{yf}$, $u_{yS}$ and $u_{ym}$ are the particle displacements along the *y*-axis on *x-z* plane in first waveguide layer, substrate and second waveguide layer, respectively, and their general solution forms can be defined as below.

(4) $$\begin{array}{l} u_{yf}=\left(Ae^{-i\beta_{f}z}+Be^{i\beta_{f}z}\right)e^{i(kx-\omega t)}\\ u_{yS}=\left(Ce^{-\beta_{S}z}+De^{\beta_{S}z}\right)e^{i(kx-\omega t)}\\ u_{ym}=\left(Ee^{-i\beta_{m}z}+Fe^{i\beta_{m}z}\right)e^{i(kx-\omega t)} \end{array}\;\mathrm{where}\;\begin{array}{l} \beta_{f}=\sqrt{\frac{\omega^{2}}{V_{f}^{2}}-k^{2}}\\ \beta_{S}=\sqrt{k^{2}-\frac{\omega^{2}}{V_{S}^{2}}}\\ \beta_{m}=\sqrt{\frac{\omega^{2}}{V_{m}^{2}}-k^{2}} \end{array}$$

The *A*, *B*, *C*, *D*, *E* and *F* are the coefficients to be solved, and *k* is the wave number.

### 2.2. Boundary Condition

The boundary conditions can be classified into two types. The first one is at the boundary between the two different materials. Both the particle displacements and stress are equal at the boundaries. The second one is the stress which is equal to zero at both the surface and bottom of the device. Thus, the boundary conditions for the monolayer and double-layer Love wave sensors can be defined as Equations (5)–(8) [26,27].

(5) $$\begin{array}{c} u_{yS}=u_{yf}\\ \mu_{S}\frac{\partial u_{yS}}{\partial z}=\mu_{f}\frac{\partial u_{yf}}{\partial z} \end{array}\;\mathrm{when}\;z=0$$

(6) $$\frac{\partial u_{yf}}{\partial z}=0\;(\mathrm{monolayer})\;\begin{array}{c} u_{ym}=u_{yf}\\ \mu_{m}\frac{\partial u_{ym}}{\partial z}=\mu_{f}\frac{\partial u_{yf}}{\partial z} \end{array}\;(\text{double-layer})\;\text{when}\;z=h$$

(7) $$\frac{\partial u_{ym}}{\partial z}=0\;(\text{double-layer})\;\text{when}\;z=h+d$$

(8) $$\mu_{S}\frac{\partial u_{yS}}{\partial z}=0\;\text{when}\;z\to -\infty$$

### 2.3. Dispersion Equation

Substituting the boundary conditions into the particle displacement equations, the system of the homogeneous linear equations of the monolayer structure can be obtained as shown in Equation (9).

(9) $$\begin{array}{l} A+B-D=0\\ -A+B+i\xi D=0\\ A-e^{i2\beta_{S}h}B=0 \end{array}\;\text{where}\;\xi =\frac{\mu_{S}\beta_{S}}{\mu_{f}\beta_{f}}$$

The condition for this system having nontrivial solutions is that the value of its coefficient determinant is zero. Using Euler's formula, the Love wave dispersion equation can be obtained as Equation (10). This dispersion equation shows the relationship among the Love wave velocity, the waveguide layer thickness and the material parameters of the substrate and waveguide layers.

(10) $$\tan(\beta_{S}h)=\xi$$

Similarly, the coefficient matrix of the double-layer structure is described as:

(11) $$\Gamma =\left(\begin{array}{ccccc} 1 & 1 & -1 & 0 & 0\\ -1 & 1 & i\xi & 0 & 0\\ e^{-i\beta_{f}h} & e^{i\beta_{f}h} & 0 & -e^{-i\beta_{m}h} & -e^{i\beta_{m}h}\\ -e^{-i\beta_{f}h} & e^{i\beta_{f}h} & 0 & \xi_{m}e^{-i\beta_{m}h} & -\xi_{m}e^{i\beta_{m}h}\\ 0 & 0 & 0 & -e^{-i\beta_{m}(h+d)} & e^{i\beta_{m}(h+d)} \end{array}\right)$$

When the determinant |Γ|=0, the dispersion equation of the double-layer Love wave sensor can be obtained as Equation (12). It is indicated that the Love wave velocity of the double-layer substrate is also affected by the thickness and material parameters of the second waveguide layer.

(12) $$\xi_{m}\tan\beta_{m}d=-\frac{\tan\beta_{f}h-\xi }{\xi \tan\beta_{f}h+1}\;\text{where}\;\xi_{m}=\frac{\mu_{m}\beta_{m}}{\mu_{f}\beta_{f}}$$

### 2.4. Particle Displacement and Wave Energy

By solving the fundamental solutions of the homogeneous linear equations system, the normalized displacements of the monolayer structure can be calculated by Equation (13).

(13) $$\begin{array}{l} u_{yf}=\frac{\cos(\beta_{f}(z-h))}{\cos(\beta_{f}h)}e^{i(kx-\omega t)}\\ u_{yS}=e^{\beta_{S}z}e^{i(kx-\omega t)} \end{array}$$

The normalized displacements of the double-layer structure can be described as:

(14) $$\begin{array}{c} u_{yf}=\left(\cos\beta_{f}z+\xi \sin\beta_{f}z\right)e^{i(kx-\omega t)}\\ u_{yS}=e^{\beta_{S}z}e^{i(kx-\omega t)}\\ u_{ym}=\frac{a\cos\beta_{m}(z-h-d)}{\cos\beta_{m}d}e^{i(kx-\omega t)} \end{array}\;\text{where}\;a=\cos\beta_{f}h+\xi \sin\beta_{f}h$$

The total time-averaged kinetic energy in the substrate ($W_{kS}$), the first waveguide layer ($W_{kf}$) and the second waveguide layer ($W_{km}$) can be calculated as [26]:

(15) $$\begin{array}{l} W_{kS}=\frac{1}{4}\omega^{2}\rho_{S}\int_{-\infty }^{0}{\left|u_{yS}\right|}^{2}dz\\ W_{kf}=\frac{1}{4}\omega^{2}\rho_{f}\int_{0}^{h}{\left|u_{yf}\right|}^{2}dz\\ W_{km}=\frac{1}{4}\omega^{2}\rho_{m}\int_{h}^{d}{\left|u_{ym}\right|}^{2}dz \end{array}$$

Substituting the particle displacements of Equations (13) and (14) into Equation (15), respectively, the wave energy in the monolayer Equation (16) and double-layer Equation (17) Love wave sensor can be calculated. Further, ${\left|A\right|}^{2}$ is the normalized coefficient.

(16) $$\begin{array}{c} W_{kS}=\frac{1}{8}\omega^{2}{\left|A\right|}^{2}\rho_{S}/\beta_{S}\\ W_{kf}=\frac{1}{8}\omega^{2}\rho_{f}{\left|A\right|}^{2}\left(\beta_{f}h+\sin(\beta_{f}h)\cos(\beta_{f}h))/(\beta_{f}\cos^{2}(\beta_{f}h)\right) \end{array}$$

(17) $$\begin{array}{c} W_{kS}=\frac{1}{8}\omega^{2}{\left|A\right|}^{2}\rho_{S}/\beta_{S}\\ W_{kf}=\frac{1}{8}\omega^{2}\rho_{f}{\left|A\right|}^{2}\left(\left(1+\xi^{2})\beta_{f}h+(1-\xi^{2})\sin(\beta_{f}h)\cos(\beta_{f}h)-\xi (\cos(2\beta_{f}h)-1\right)\right)/\beta_{f}\\ W_{km}=\frac{1}{8}\omega^{2}\rho_{m}a^{2}{\left|A\right|}^{2}\left(\beta_{m}d+\sin(\beta_{m}d)\cos(\beta_{m}d)\right)/\left(\beta_{m}\cos^{2}(\beta_{m}d)\right) \end{array}$$

### 2.5. Sensitivity of the Love Wave Sensor

The sensitivity of the Love wave sensor can be calculated based on the perturbation theory [5,28]. The mass loading on the surface can be considered as a small perturbation causing a small change of the kinetic energy, which can be obtained through the particle displacements and energy transport [26]. It is known that with the increasing waveguide layer thickness, more energy is present in the waveguide layer than in the substrate. It can be approximated that this is similar to the variation of the energy repartition in the structure when a mass loading film attaches to the surface [29]. The mass loading (density $\rho_{l}$, thickness Δε) is a very thin film in comparison to the waveguide layer, and thus it can be considered as a slight thickness increase of the waveguide layer, which can be considered as $\rho_{l}\Delta \varepsilon =\rho_{f}\Delta h$. Hence, the mass loading sensitivity can be estimated by the energy variation in the substrate to the mass modification via Equation (18).

(18) $$S_{m}=\frac{1}{W}\underset{\Delta m_{l}\to 0}{\lim}\frac{\Delta W}{\Delta m_{l}}\approx \frac{1}{\rho_{f}\lambda W}\frac{dW}{d\alpha }=\frac{K}{\rho_{f}\lambda }$$

The normalized thickness is α=h/λ, and λ is the wavelength of the Love wave. With the same method for the sensitivity calculation of the monolayer Love wave sensor, the mass loading sensitivity of the double-layer Love wave sensor can be described as:

(19) $$S_{m}\approx \frac{1}{\rho_{m}\lambda W}\frac{dW}{d\alpha }=\frac{K}{\rho_{m}\lambda }$$

As the density and the wavelength are constant coefficients for certain Love wave devices, the non-dimensional factor *K*, named the sensitivity coefficient, can be used to describe the sensitivity of different devices with various wavelengths.

## 3. Results and Discussion

### 3.1. Results of Love Wave Velocity

**Figure 2:**
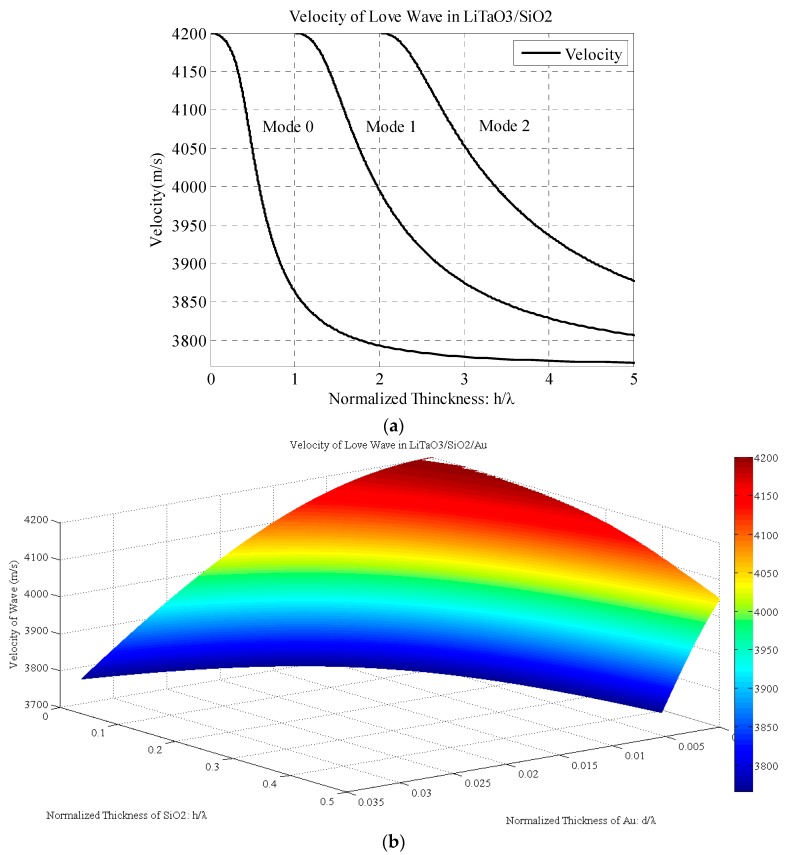
Velocity of Love wave in (**a**) LiTaO_3_/SiO_2_ device; (**b**) LiTaO_3_/SiO_2_/Au device.

As the dispersion equations (Equations (10) and (12)) are implicit functions and cannot be calculated directly, we calculated them using MATLAB. The monolayer Love wave velocity is shown in Figure 2a. It is clearly indicated that increasing the layer thickness decreases the velocity of the Love wave from the shear velocity in the substrate to that in the waveguide layer. The multi-modes of the Love wave are owing to the multiple solutions of the trigonometric function. Generally, the thickness of the waveguide is very thin compared with the wavelength, and thus only the 0-mode should be considered.

The velocity of the Love wave in the LiTaO_3_/SiO_2_/Au structure is shown in Figure 2b. It is indicated that the velocity decreases from the velocity of the LiTaO_3_ substrate when the thicknesses of both the SiO_2_ and Au waveguide layer are increasing. The contour plot shows that even when the SiO_2_ or Au layer thickness is constant, the velocity of the Love wave decreases with the other layer thickness increasing. Owing to the lower velocity, the Love wave velocity decreases much faster in the Au layer direction than in the SiO_2_ direction.

### 3.2. Results of Love Wave Energy

**Figure 3:**
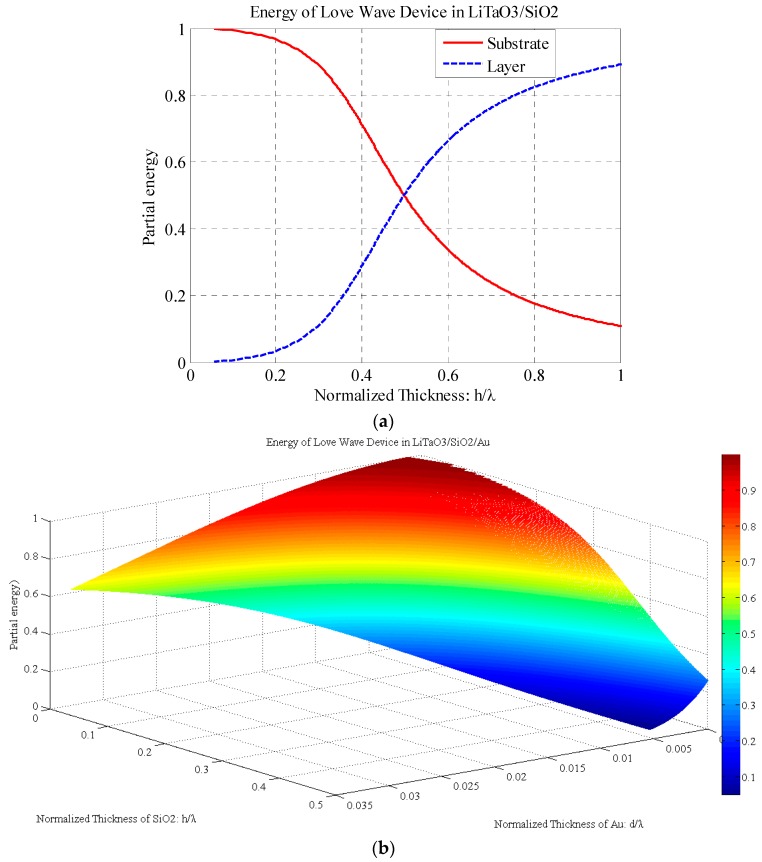
(**a**) Partial energy of Love wave in LiTaO_3_/SiO_2_ device; (**b**) Partial energy of substrate in LiTaO_3_/SiO_2_/Au device.

After obtaining the functional relationship between the velocity and waveguide layer thickness, the total time-averaged kinetic energy in the substrate and waveguide layers can be solved according to Equations (16) and (17). As seen explicitly in Figure 3a, the partial energy in the substrate and that in the waveguide layer varies with the thickness; however, the total energy of the Love wave is assumed to be constant. At the beginning, most of the wave energy propagates in the substrate. With the thickness of the waveguide layer increases, the energy moves from the substrate to the waveguide layer, leading to highly centralized energy in the waveguide layer.

Figure 3b shows the partial energy proportion in the LiTaO_3_ substrate. The energy in the substrate decreases with both the SiO_2_ and Au waveguide layer thickness increasing. It is indicated that more wave energy moves from the substrate to the waveguide layer. The decreasing rates in the SiO_2_ and Au layer directions are different because of their different material parameters.

### 3.3. Results of Love Wave Sensors’ Sensitivity

**Figure 4:**
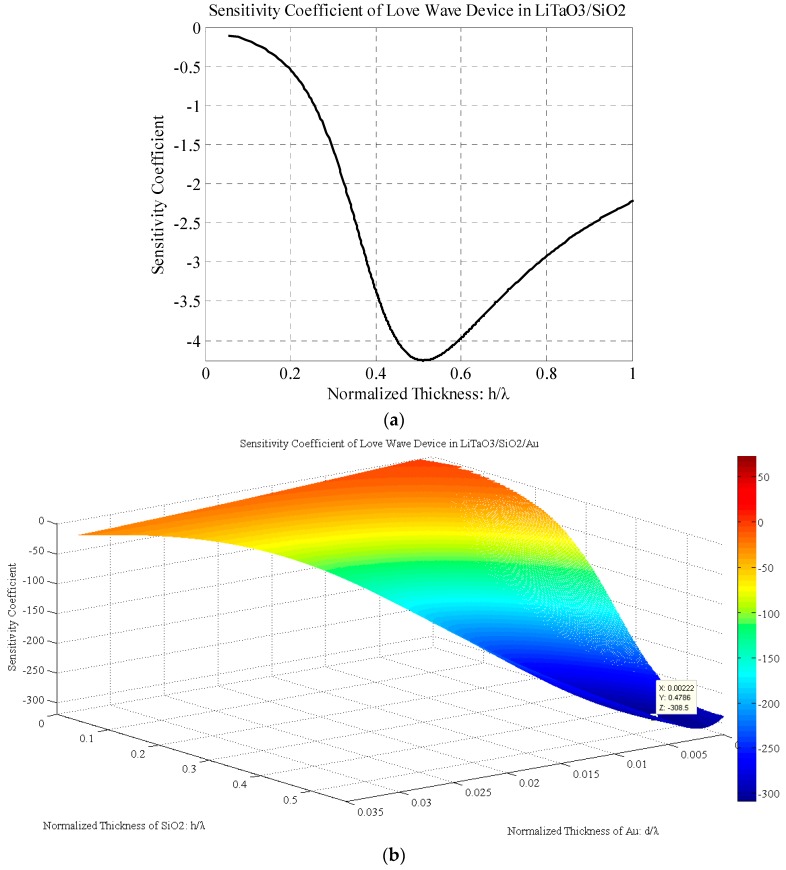
Sensitivity coefficient of Love wave devices: (**a**) LiTaO_3_/SiO_2_ structure; (**b**) LiTaO_3_/SiO_2_/Au structure.

The sensitivity coefficients of the monolayer and double-layer structures can be calculated via the energy variation in the substrate. The sensitivity coefficient of the LiTaO_3_/SiO_2_ Love wave sensor is shown in Figure 4a. Initially the absolute value of the sensitivity coefficient increases when the SiO_2_ layer thickness increases, and it reaches the optimal value (*K* = −4.25) at the normalized thickness of 0.51. Then the sensitivity coefficient decreases with the layer thickness increasing.

As shown in Figure 4b, the sensitivity coefficient of the LiTaO_3_/SiO_2_/Au Love wave sensor reaches an optimal value (*K* = −308.4656) when the normalized thicknesses of the SiO_2_ and Au layers are at 0.4786 and 0.0022, respectively. The 3D contour plot reveals the higher sensitivity coefficient of the LiTaO_3_/SiO_2_/Au structure compared to that of the monolayer structure. By using the SiO_2_/Au as a double-layer waveguide, the sensitivity coefficient of the Love wave sensor is enhanced about 72-fold. 

Comparing the monolayer and double-layer Love wave sensors, the optimal thickness condition of the SiO_2_ layer is decreased by the added Au layer. Using 20 µm as the wavelength of the Love wave, for example, the optimal SiO_2_ thickness is found to be 10.2 µm for the monolayer Love wave sensor and 9.57 µm for the double-layer one. The optimal thickness of Au is only about 44 nm, and this thin layer is very easy to be fabricated using Au deposition. Even for the decreased SiO_2_ thickness layer range of about 1–4 µm, the sensitivity coefficient of the double-layer sensor is still higher than that of the monolayer one. For example, the sensitivity coefficient is equal to −100 when the SiO_2_ layer and Au layer are 4 µm and 74 nm, respectively. As a result, this leads to a much easier fabrication and a lower insertion loss compared with the monolayer sensors with a SiO_2_ layer thicker than 10 µm.

## 4. Conclusions

In this work, the theoretical analysis of the monolayer and double-layer waveguide Love wave sensors is presented. The variations of the energy repartition in the structure are utilized to calculate the sensitivity coefficients of the Love wave sensors. It is indicated that the optimized normalized thickness of the SiO_2_ layer for the monolayer 36° YX LiTaO_3_ substrate is 0.51. A 72-fold enhancement of the sensitivity coefficient is achieved by using a SiO_2_/Au double-layer structure, when the optimal normalized thicknesses are 0.4786 and 0.0022, respectively. In addition, the thickness of the SiO_2_ layer to achieve the same and an even higher sensitivity coefficient as that of the monolayer sensor is significantly reduced with the help of the thin gold layer. Our results provide guidelines for the design and fabrication of Love wave sensors to achieve a high sensitivity and to reduce the device fabrication difficulties.

## Figures and Tables

**Table 1:** Parameters of different materials.

Materials	Density (kg/m^3^)	Shear Velocity (m/s)
SiO_2_	2200	3766
Au	19,300	1215
36° YX LiTaO_3_	7454	4200
